# Comparative population genomic analysis uncovers novel genomic footprints and genes associated with small body size in Chinese pony

**DOI:** 10.1186/s12864-020-06887-2

**Published:** 2020-07-20

**Authors:** Hojjat Asadollahpour Nanaei, Ali Esmailizadeh, Ahmad Ayatollahi Mehrgardi, Jianlin Han, Dong-Dong Wu, Yan Li, Ya-Ping Zhang

**Affiliations:** 1 Department of Animal Science, Faculty of Agriculture, Shahid Bahonar University of Kerman, Kerman, PB 76169-133 Iran; 2 State Key Laboratory of Genetic Resources and Evolution and Yunnan Laboratory of Molecular Biology of Domestic Animals, Kunming Institute of Zoology, Chinese Academy of Sciences, No. 32 Jiaochang Donglu, Kunming, Yunnan China; 3 CAAS-ILRI Joint Laboratory on Livestock and Forage Genetic Resources, Institute of Animal Science, Chinese Academy of Agricultural Sciences (CAAS), Beijing, China; 4 Livestock Genetics Program, International Livestock Research Institute (ILRI), Nairobi, Kenya; 5 State Key Laboratory for Conservation and Utilization of Bio-Resources in Yunnan and Center for Life Sciences, School of Life Sciences, Yunnan University, Kunming, China

**Keywords:** Body size, Artificial selection, Population genomics, Horse, *NELL1*

## Abstract

**Background:**

Body size is considered as one of the most fundamental properties of an organism. Due to intensive breeding and artificial selection throughout the domestication history, horses exhibit striking variations for heights at withers and body sizes. Debao pony (DBP), a famous Chinese horse, is known for its small body size and lives in Guangxi mountains of southern China. In this study, we employed comparative population genomics to study the genetic basis underlying the small body size of DBP breed based on the whole genome sequencing data. To detect genomic signatures of positive selection, we applied three methods based on population comparison, fixation index (*F*_ST_), cross population composite likelihood ratio (XP-CLR) and nucleotide diversity (θπ), and further analyzed the results to find genomic regions under selection for body size-related traits.

**Results:**

A number of protein-coding genes in windows with the top 1% values of *F*_ST_ (367 genes), XP-CLR (681 genes), and log_2_ (θπ ratio) (332 genes) were identified. The most significant signal of positive selection was mapped to the *NELL1* gene, probably underlies the body size and development traits, and may also have been selected for short stature in the DBP population. In addition, some other loci on different chromosomes were identified to be potentially involved in the development of body size.

**Conclusions:**

Results of our study identified some positively selected genes across the horse genome, which are possibly involved in body size traits. These novel candidate genes may be useful targets for clarifying our understanding of the molecular basis of body size and as such they should be of great interest for future research into the genetic architecture of relevant traits in horse breeding program.

## Background

For thousands of years, enormous variety of domestic breeds with different morphological, physiological and behavioral characters have been domesticated and raised in different parts of the world. They therefore offer a powerful source of biological models for the biology studies and have played significant role in developmental, evolutionary and biomedical research [1–3].

Due to intensive breeding and artificial selection throughout the domestication history, horses exhibit striking variation of height at withers and body size. Today, body size is one of the most important criteria for the evaluation and classification of different breeds, and also is an essential parameter for breeding programmers to improve marketability and performance [4]. According to this criterion, most horse breeds can be divided into three main categories including high stature/heavy horses (draft breeds), light horse (riding breeds) and small stature/low weight (pony breeds) [5, 6].

The pony breed is defined as a group of horses with a common height less than 14.2 hands (147 cm) at the withers [7]. There are several pony breeds in the world, though varying in body size, skin color and geographic origins, all of them share some basic traits that make them different from other horse breeds. Debao pony (DBP), a famous Chinese horse breed, is known for its small body size and lives in Guangxi mountains of southern China. This breed is strictly protected by the Chinese government, therefore it has the largest population compared with other local pony breeds. DBPs exhibit peculiar morphoanatomical adaptation to facilitate work in the mountainous regions, for example their average adult height is around 94 cm and 98 cm for male and females, respectively [8, 9].

Although previous studies reported a few candidate genes such as *LCORL*, *HMGA2*, *ZFAT*, *NCAPG* [6, 10], *TBX3* [9] and *ANKRD1* [11] with major effects on height and body size variations in several horse breeds, these studies were mostly carried out using SNP genotyping involving Illumina EquineSNP50 Genotyping Beadchip or GeneSeek Equine SNP70 Beadchip, which certainly have some limitation due to factors such as low SNP density in many genomic regions, differential probe affinity as well as ascertainment biases that prevent them from detecting novel variation in entire genome [12].

In the present study, whole genome sequencing (WGS) data were used for comparative population genomics to identify the genetic basis underlying the small stature of DBP.

## Results

### Sequencing and read alignment

Individual genomes of 17 DBPs were sequenced on an Illumina Hiseq 2000 platform with a read length of 125 bp. To facilitate comparisons with other horse breeds, the sequenced horse data were jointly analyzed with publicly available WGS data of 15 different breeds (*n* = 69) (Additional file 1: Table S1 and Additional file 2: Figure S1). The mean sequence depth was ~ 13.5X per sample (Additional file 2: Figure S2). We detected a total of 18,384,176 SNPs in all individuals (Additional file 1: Table S2).

### Phylogenetic analysis, runs of homozygosity and linkage disequilibrium decay

**Fig. 1:**
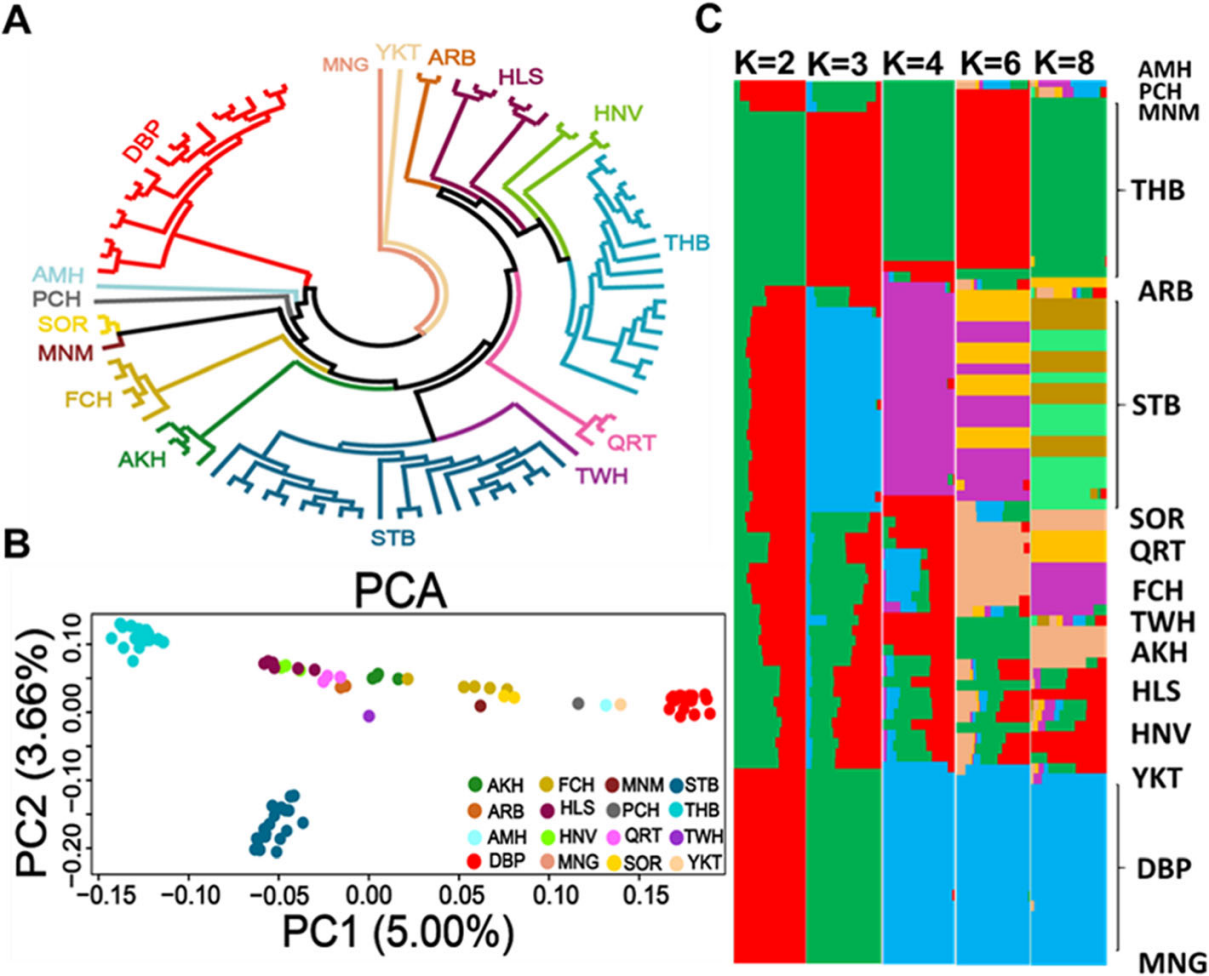
Phylogenetic analyses. **a** Phylogenetic tree was built based on weighted neighboring-joining (NJ) method, using Mongolian horse as outgroup. **b** PCA. **c**. Population structure by Admixture program with K = 2 to 8 (K = 2 best). AKH, Akhal-Teke; ARB, Arabian; AMH, American Miniature Horse; DBP, Debao pony; FCH, Franches-Montagnes; HLS, Holsteiner; HNV, Hanoverian; MNG, Mongolian; MNM, Mangalarga Marchador; PCH, Percheron; QRT, Quarter Horse; SOR, Sorraia; STB, Standardbred; THB, Thoroughbred; TWH, Tennessee Walking Horse and Yakutian, YKT

**Fig. 2:**
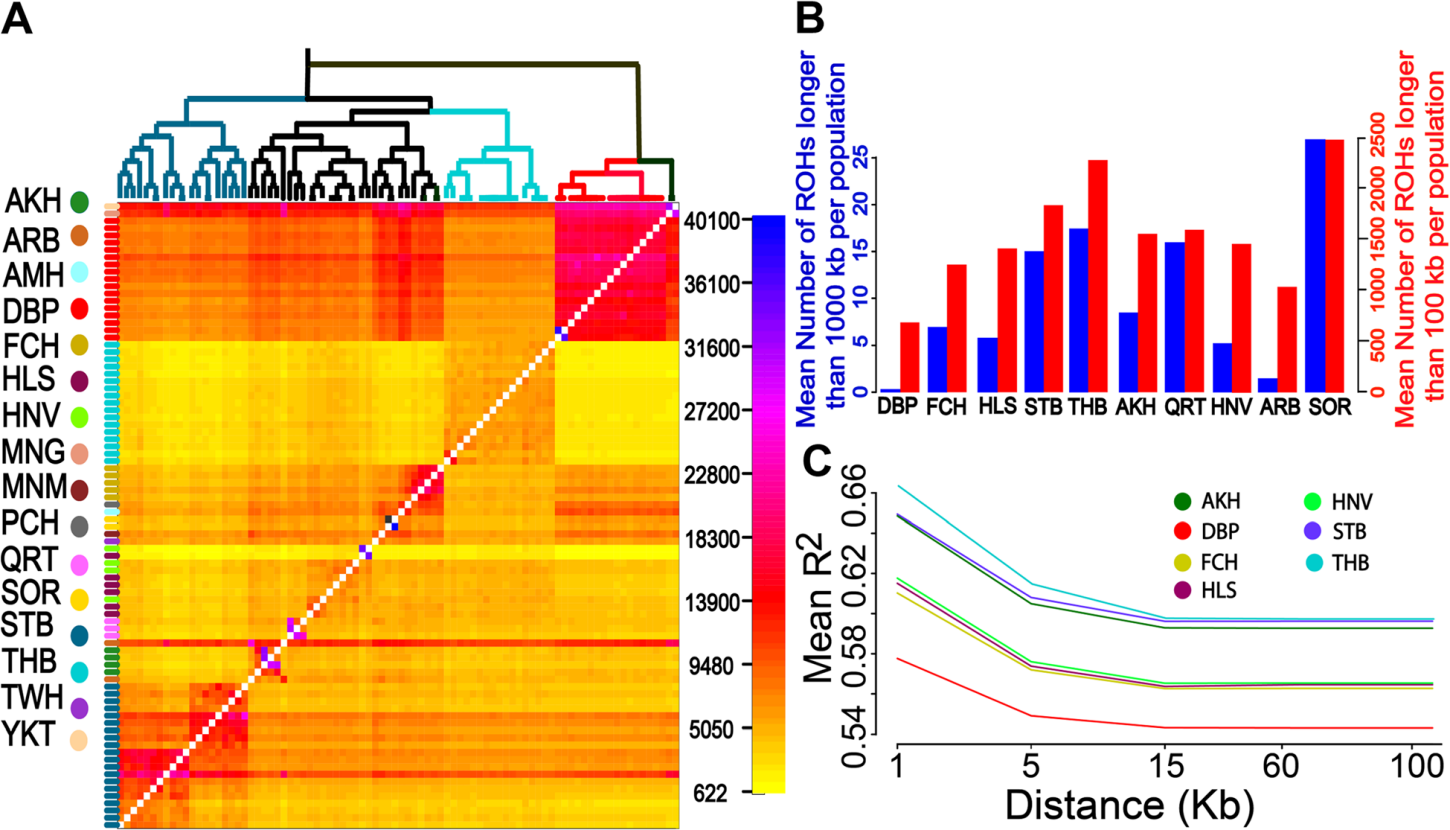
**a** Finestructure. A heat map of a co-ancestry matrix generated by chromosome painting with fineSTRUCTURE. The color of each cell represents the expected number of ‘chunks’ imported from a donor genome (column) to a recipient genome (row). **b** Runs of homozygosity (ROH). **c** Linkage disequilibrium (LD) decay

We firstly performed different classical analyses including phylogenetic tree, principal component analysis (PCA), Bayesian model-based analysis and haplotype population structure based on chromosome painting from ChromoPainter and fineSTRUCTURE analyses to reveal genetic relationships among different horses (Figs. 1 and 2a). Based on the phylogenetic tree results (Fig. 1a), all DBPs were separated from Middle East, European and American horse breeds. The topological pattern found in the tree was also supported by both PCA (Fig. 1b) and Bayesian model-based analysis (Fig. 1c and Additional file 2: Figure S3). The results of painting algorithm from ChromoPainter and fineSTRUCTURE analyses simplified their relationships into a co-ancestry matrix, which presents expected number of chunks from donors (column) to recipients (row), and visualized as a heat map plot (Fig. 2a).

In addition, runs of homozygosity (ROH) along the whole genome have been applied to quantify individual autozygosity to improve the understanding of inbreeding depression in populations. The mean number of ROHs longer than 100 and 1000 Kb per each population is plotted in Fig. 2b. Also, to investigate the effects caused by selection and genetic bottleneck on population history and demography, we determined the scale of linkage disequilibrium (LD) decay for each breed. The average level of LD, measured by *r*^2^, between adjacent single-nucleotide polymorphism (SNPs) across the complete genome was estimated (Fig. 2c).

### Scans for signatures of selection

Based on the results obtained from phylogenetic analysis and to avoid bias from unequal sample sizes, we compared the whole genome of DBP individuals, as a pony-sized (genetically homogeneous) breed with Thoroughbred (THB) horses, as a large well-defined (genetically homogeneous) breed, to identify signatures of positive selection related to body size traits. Here, we examined three different parameters for a greater statistical power to localize the source of selection signals. Population differentiation (*F*_ST_) was calculated for each SNP between DBP and THB horses as described in Weir and Cockerham [13]. A sliding window analysis was employed with 50 kb window size and 25 kb step size. A total of 367 protein-coding genes were identified in the top 1% windows with high *F*_ST_ values (Additional file 1: Table S3). To get a clearer insight into the genetic mechanisms related to these candidate genes, further downstream analyses were conducted. Among the candidate genes, five (*NELL1*, *FGFR1*, *SNTG1*, *BMP2* and *TBX15*) are involved in body size related traits (Table 1). Gene set enrichment analysis (GSEA) identified some significantly enriched categories related with skeletal development, such as “broad hallux” (HP:0010055), “broad phalanges of the hand” (HP:0009768) and “broad toe” (HP:0001837) (Additional file 1: Table S4). We then, using a log_2_ ratio of θπ between DBP and THB samples, identified genomic regions that may have been under selection in DBPs. A sliding window analysis yielded 332 candidate genes (Additional file 1: Table S5). For these extreme values, some enriched gene ontology (GO) terms were found to be related with skeletal development categories, including “aplasia/hypoplasia of the tibia” (HP:0005772) and “Short femur” (HP:0003097) (Additional file 1: Table S6). In addition, we found some size-related genes such as, *NELL1*, *FGFR1* and *CNN3* (Table 1). Finally, we adopted a cross-population composite likelihood ratio test (XP-CLR) to evaluate historical selections based on the comparison of allele frequency spectrum. A total of 681 candidate genes were identified in the top percentiles of approach (1% cutoff) (Additional file 1: Table S7). The results of functional enrichment analysis from all these genes showed some categories related with both muscle and skeletal development such as “small hand” (HP:0200055), “increased body weight” (HP:0004324), “large forehead” (HP:0002003), “animal organ development” (GO:0048513), “anatomical structure development” (GO:0048856) and “tissue development” (GO:0009888) (Table 1; Additional file 1: Table S8).

**Table 1:** Candidate genes putatively selected by three statistical methods (*F*_ST_, log_2_ θπ ratio and XP-CLR) affecting body size traits in DBP

Method	Gene	Chr.^a^	Ensembl ID	Summary of gene function
*F*_ST_ (top 1%)	*NELL1*	7	ENSECAG00000024835	Cell differentiation and cell proliferation [14, 15], Short stature [16]
	*FGFR1*	27	ENSECAG00000015006	Bone growth and skeletal development [17–20]
	*SNTG1*	9	ENSECAG00000000087	Body measurement traits [21]
	*BMP2*	22	ENSECAG00000021201	Bone physiology and metabolism [22–25].
	*TBX15*	5	ENSECAG00000023325	Skeletal muscle and muscle metabolism [26]
log2(θπ·DBP/θπ·THB) (top 1%)	*NELL1*	7	ENSECAG00000024835	Cell differentiation and cell proliferation [14, 15], Short stature [16]
	*FGFR1*	27	ENSECAG00000015006	Bone growth and skeletal development [17–20]
	*TRHDE*	28	ENSECAG00000009284	Growth Traits [27]
	*CAPN7*	16	ENSECAG00000004932	Growth Traits [28]
	*GALNT10*	14	ENSECAG00000006979	Body mass index [29]
	*PDE1B*	6	ENSECAG00000022552	Muscle growth [30]
	*ARPP21*	16	ENSECAG00000022872	Body size traits [31]
	*FAM210A*	8	ENSECAG00000007436	Bone and muscle structure [32]
	*CNN3*	5	ENSECAG00000020188	Skeletal muscle development [33]
	*SWT1*	5	ENSECAG00000012085	Carcass weight [34]
	*PRDM16*	2	ENSECAG00000017045	Body weight [34]
XP-CLR (top 1%)	*NELL1*	7	ENSECAG00000024835	Cell differentiation and cell proliferation [14, 15], Short stature [16]
	*SNTG1*	9	ENSECAG00000000087	Body measurement traits [21]
	*BMP2*	22	ENSECAG00000021201	Bone physiology and metabolism [22–25].
	*TRHDE*	28	ENSECAG00000009284	Growth Traits [28]
	*IGF2BP2*	19	ENSECAG00000020685	Embryonic development [35]
	*PRKG2*	3	ENSECAG00000024387	Dwarfism [36]
	*ADAMTS17*	1	ENSECAG00000000579	Human height [37], Development of body size in horses [37]
	*SH2B2*	13	ENSECAG00000024201	Growth performance [38]
	*PLXDC2*	29	ENSECAG00000017520	Body size traits [39]
	*TNS3*	4	ENSECAG00000020052	Bone length [39]
	*AGTPBP1*	23	ENSECAG00000019812	Body mass index [40]

^a^Chromosome

In addition, to confirm these results, when we compared the genome of DBP, used as a test population, with all other horse studied breeds (mixed-breed and purebred) used as a reference population, we also observed that *NELL1* signals in the high values (windows with the top 1% values of both *F*_ST_ and XP-CLR methods, Additional file 2: Figure S4).

## Discussion

Before to the application of methods for detecting signatures of selection, we first assessed the phylogeny of the horse breeds to evaluate the phylogenetic position of DBP within the species. In agreement with previous studies, DBP was phylogenetically distant from other horse breeds while being relatively close to the Mongolian horse (MNG) [8, 9]. The distance patterns between horse breeds was also identified by PCA. However, THB and DBP had the greatest distance from each other, Hanoverian (HNV) and Holsteiner (HLS) breeds were not clearly distinguishable in either the phylogenetic tree and PCA, indicating the possibility of shared genetic components between these two German Warmblood breeds [41]. Similar to the results from PCA, admixture analysis at *K* = 2 separated the DBP, Yakutian (YKT), MNG and THB horses from other populations, while at *K* = 3 the Standardbred (STB) horses separate from the remaining horse populations. Consistent with previous studies, we found that the DBP and THB populations are genetically homogeneous for all *K* values (*K* = 2 to *K* = 8) [4, 9].

In addition, LD decay analysis revealed a markedly lower level of LD across all genomic distances in DBPs than other breeds. The high LD in commercial breeds, especially for THB horses, could be a consequence of artificial selection for specific abilities, e.g. racing performance, in the breeding programs [42] while DBPs clearly have an ancient origin following a long-term natural selection. Also, discovering the ROH per each breed showed the lower level of ROH in the DBP population compared to other horses. Here, we found high level of ROH for THB population, that is concordant with previous study in the six different horse breeds [43].

### Potential independent of positive selection in the DBP population

**Fig. 3:**
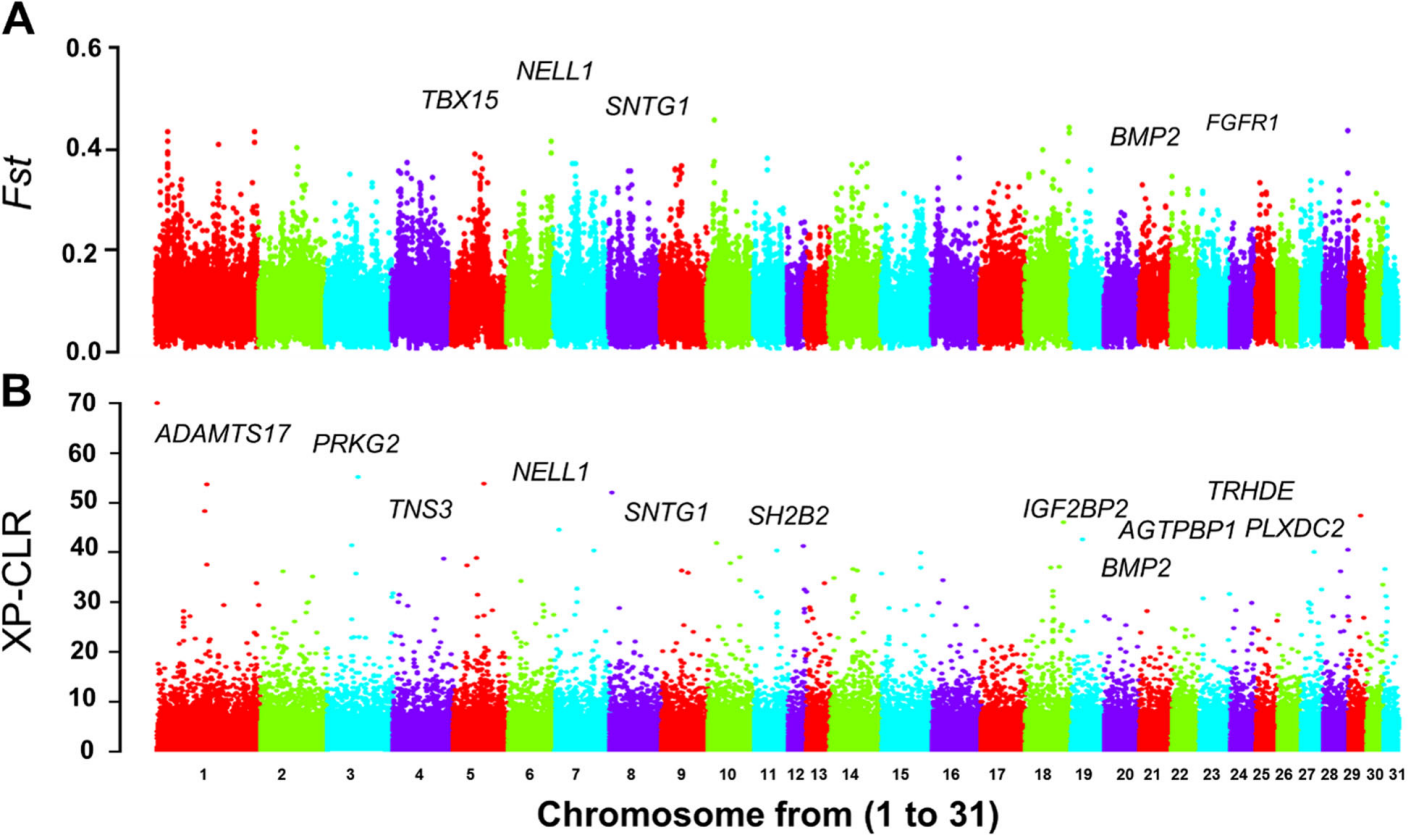
Genomic landscape of population differentiation by *F*_ST_ (**a**) and XP-CLR (**b**)

Because of the small body size of DBP, that is notably less than average horse breeds, we focused specifically on the loci that may play more important roles in the rapid evolution of body size during the domestication process. Here, we used comparative genome analysis between DBP and THB breeds to identifying the genetic basis underlying the size variation among DBPs. In our broad spectrum analysis, several previously reported genes were found to be probably involved in body size related traits. Highly significant candidate genes related to these traits are listed in Table 1. Within the regions showing extremely high values (top 0.01), both *F*_ST_ and XP-CLR methods showed *BMP2* gene shared overlapped selection signatures as positively selected genes (PSGs) (Fig. 3a and b). *BMP2*, a bone formation-related gene, was found as one of the candidates on ECA5. This protein belongs to the TGF-β superfamily, which has diverse biological activities related to bone physiology and metabolism [22, 23]. Previous studies have found associations of the *BMP-2* variants with bone and cardiac development, bone mineral density, as well as body size traits [24, 25]. Also in human, BMP-2 appears to be the most important BMP affecting the adult skeleton [44].

Another possible candidate gene, *FGFR1*, was found in one of the selection regions on ECA27 (top 1% cutoff for *F*st and log2 θπ ratio values) (Fig. 3a)*. FGFR1* is an important candidate gene that influences bone growth and skeletal development. Previous studies found that FGFR1 protein plays a critical role in formation of muscle and bone tissues [17–20]. Considering the important function of *FGFR1* in skeletal development, this gene is an important candidate for body size variation in mammalians.

**Fig. 4:**
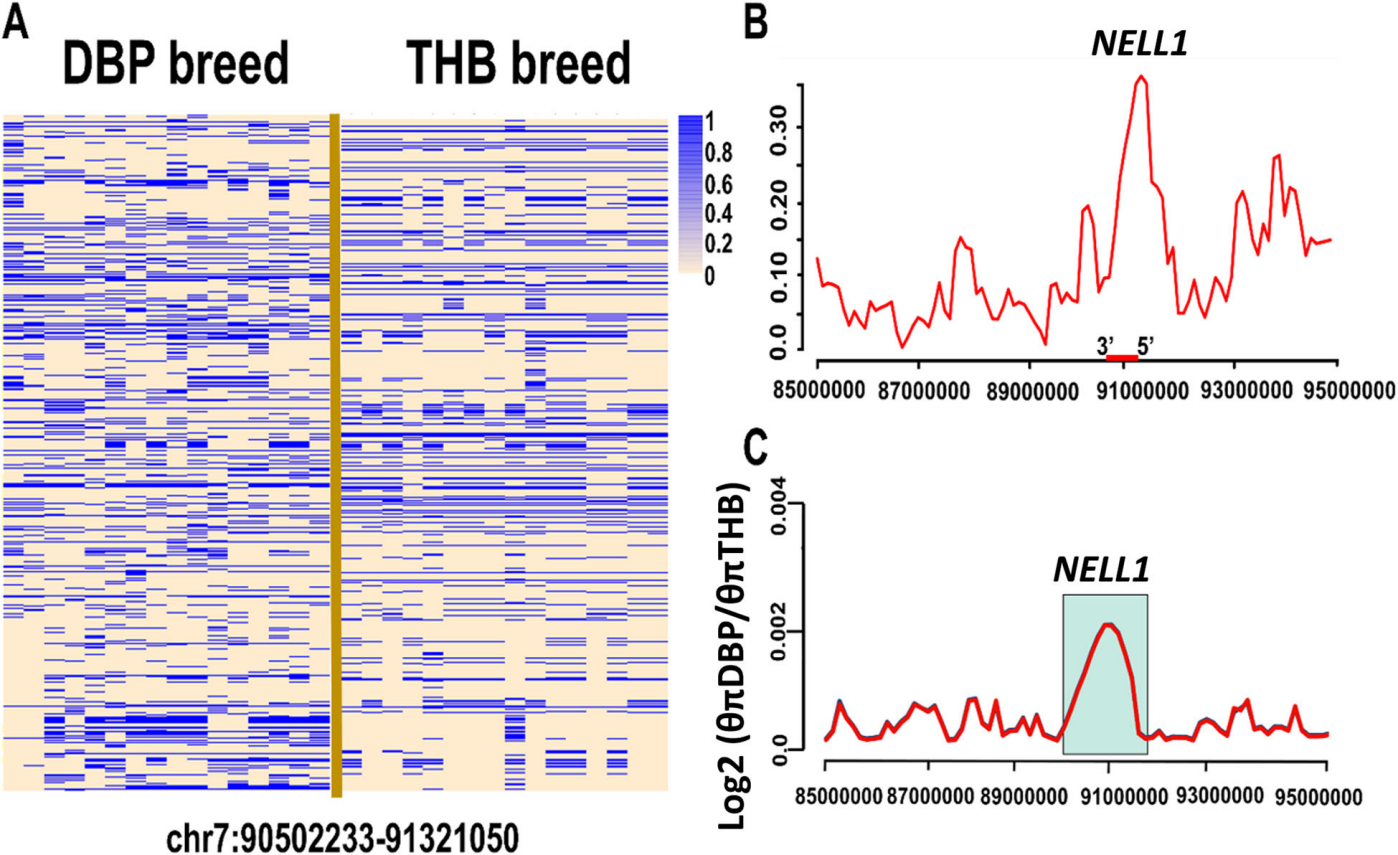
Positive selection on *NELL1* gene, (**a**) haplotype across *NELL1* gene, (**b**) *F*_ST_ and (**c**) log2 (θπ·DBP/θπ·THB)

Results from the detection of selection signatures revealed consistently high signal values in *F*_ST_ and XP-CLR analyses as well as log2 θπ ratio for *NELL1* gene, which is overlapped among candidate PSGs (Figs. 3a, b and 4). *NELL1*, encodes a mammalian cell-signaling protein (protein kinase C-b1, PKC-b1) that has been shown to regulate skeletal ossification [45, 46]. Overexpression of this gene in both human and mice induces craniosynostosis, the premature fusion of cranial sutures [14]. Previous studies have shown that absence of *Nell1* leads to decreased cell differentiation and cell proliferation in several organs such as heart, bone and cartilage tissue [14, 15]. Recently, an interstitial 11p14.1-p15.3 deletion involving the *Nell-1* gene was also reported in associated with short stature in children [16]. Moreover, it was demonstrated that the *NELL-1* has potential roles as a bone-forming growth factor in sheep [47].

Body size is recognized as one of the most fundamental properties of an organism, affecting nearly all biological aspects. In the last decades, new insights from the genetic and physiological studies have refined our understanding of genetic basis of body size, as the target of positive selection in human and domesticated animals. Human body size is a polygenic trait affected by variants of numerous genes and their interactions with environmental factors. For example, hundreds of genetic variants, in at least 180 loci, with small effects, have impact on final human adult height [48]. In contrast, several independent studies in domesticated animals have shown that changes in body size can be controlled by a few genes with large effects. For instance, it has been demonstrated that one specific haplotype defined by 20 SNPs spanning the recent selection sweep covering *IGF-1* gene has a major effect on body size within all small dogs [49]. A similar study has shown that one SNP within the strong linkage region of *BMP10* gene explained around 22% of the overall body weight variance in five chicken lines [2]. Also, one study on dairy and beef cattle revealed the variation in the average height can be controlled by only 10 genes in eight genomic regions [50].

Based on the standard additive model, Makvandi-Nejad et al. [6] identified four loci on the ECA3, 6, 9 and 11 that explained 83% of size variance in 48 horses, three each of eight large and eight small horse breeds. Using the same dataset of these 48 horses, a recent GWAS study involving both dominant and recessive mixed-model approaches as well as a genome-wide scan for signatures of selection based on the *F*_ST_ genetic differentiation and XP-CLR test, *ANKRD1* gene was identified and validated as a novel candidate, explaining 7.98% of the genetic variance in body size of the American Miniature horse (AMH). Compared with the fixed status of all four loci identified by Makvandi-Nejad et al. [6], *ANKRD1* gene could be applied in effective genotype-assisted selection for body size in AMH [11]. In other independent studies, the differential SNPs in *LCORL* gene on ECA3 [10, 51–54], *ZFAT* gene on ECA9 [51], *TBX3* gene on ECA8 [9] and *LASP1* gene on ECA11 [55] have also been shown to be strongly associated with body size traits in horses.

In this study, we have investigated the genetic basis underlying the body size variation in DBP. In our broad spectrum of analyses by three methods, we did not find any significant selection signal within or near genes which were previously identified as horse body size-related candidates. Instead, we observe that *NELL1* gene likely played an important role in the evolution of the small stature of DBP, an ancient small pony that was evolved in the mountainous areas in southwestern China. In addition, some other loci on different chromosomes were also identified to be potentially involved in body development process. No evidence of directional selection for the detected genes in this study has been reported to date in other horse populations, suggesting that these genes have been probably selected independently for the short stature of DBPs.

## Conclusions

In this study, using next-generation sequencing analysis, we identified some novel candidate genes under selection for body size traits in DBP population. This results suggest that the imaging a common evolutionary mechanism that influences patterns of genetic variation in all horse breeds is misconception as many of them were adapted for different environments and/or for various goals. These novel candidate genes may be useful target for clarifying our understanding of the molecular basis of body size and they should be of great interest for future research addressing the genetic architecture of relevant traits in horse breeding program.

## Methods

### Sample collection and sequencing

In this study, all 17 horse blood samples were collected from private farms in Debao county, Baise city, Guangxi province in south of China. The experimental animals were not anesthetized or euthanized in order to conduct this study. No horse individuals died in this study and all individuals stayed healthy after collecting blood samples. Genomic DNA was extracted using the phenol-chloroform method. Pair-end sequence data for all DBPs were generated using the Illumina Hiseq 2000. Also, previously published genome sequence data from 15 other horse breeds (*n* = 69) were obtained from the Sequence Read Archive (ncbi.nlm.nih.gov) (Additional file 1: Table S1 and Additional file 2: Figure S1).

The sample size for our experiment was calculated based on Ma et al., guidelines [56]. Ma et al., (2015) showed that a reasonable power to detect selection signatures is achieved with high marker density (> 1 SNP/kb) as obtained from sequencing, while rather small sample sizes (~ 15 diploid individuals) appear to be sufficient. The sample size of 86 animals used in our experiment has a power of at least 80% to detect the genomic signature of selection using different approaches.

### Alignments and variant identification

High-quality reads in the present study and published data were aligned against the horse reference genome, ENSEMBL (version 94) (ftp://ftp.ensembl.org/pub/release-94/fasta/equus_caballus/dna/), using Burrows-Wheeler Aligner (BWA) (https://github.com/lh3/bwa) [57]. Binary alignment map (BAM) files were imported into SAMtools [58] for sorting/merging and into Picard tools (https://broadinstitute.github.io/picard/, latest release 2.18.26) to mark duplicated reads. We then improved alignment accuracy by minimize such mismatching bases using RealignerTargetCreator, IndelRealigner, and BaseRecalibrator functions in the Genome Analysis Toolkit (GATK) 4.0.12.0 [59]. The variant calling of sequence data were handled using UnifiedGenotyper and VariantFiltration tools in GATK. The final sequencing variants were filtered to be supported by a minimum mapping quality of 25 and a minimum genotype quality of 40. Furthermore, all loci with > 2 alleles and within clusters (> 3 SNP in a 10-bp window) were removed from later analyses.

### Population variation and population genetic analyses

The phylogenetic tree for the individual genome sequences was constructed from the SNP data by using the neighbor-joining method among the horses. The PCA was performed using the software GCTA [60], after pruning the SNPs for short-range LD by PLINK software [61]. In order to visualize population structure between different horse breeds, ADMIXTURE [62] was used with an ancestor population size ranging from 2 to 8 and 10,000 iterations for each run, based on the pruned data. In addition, we used a haplotype-based approach to explore patterns of haplotype sharing using ChromoPainter and fineSTRUCTURE [63]. The decay of LD measured as the squared correlation coefficient by pairwise physical distance for each breed. ROHs were also identified via the “Runs of Homozygosity program”, implemented in PLINK software. For each population, we identified all ROHs of length longer than 100 and 1000 kb distances [61].

### Identification of candidate genes under positive selection in the DBP

Three different statistic methods were used to identify regions of the genome that have the highest signals of selective sweeps in DBPs. To characterize genetic differentiation between DBP and THB horses, pairwise *F*_ST_ values for each SNP were calculated using Weir and Cockerham method [13]. The θπ values were calculated for both DBP and THB populations. Sliding window analyses were performed upon a window size of 50 kb and a step size of 25 kb across of the genome. The average *F*_ST_ and log2 (θπ DBP/θπ THB) values of SNPs in each window were calculated. We additionally performed XP-CLR test to detect regions in the genome where the change in allele frequency at the locus almost-occurred recently. The scores were estimated using the code provided by Hua Chen (Department of Genetics, Harvard Medical School) [64]. We used the following options: sliding windows of 0.1 cM, grid size 10 k, maximum number of SNPs within each window as 300, and correlation value for 2 SNPs weighted with a cutoff of 0.99. Here, DBPs was taken to be the object population and THB horses chosen as the reference population. The regions with the XP-CLR values in the top 0.01 of the empirical distribution were designated as signal of positive selection.

### Gene set enrichment and pathway analysis

To search the possible pathways involved in the regions with top values (*F*_ST_, log2 θπ ratio and XP-CLR), candidate selective regions were annotated using the Variant Effect Predictor available at (http://asia.ensembl.org/info/docs/tools/vep/index.html). Functional enrichment analysis was done by using the ‘g:Profiler’ enrichment analysis tool, to uncover their biological functions [65]. And the *P*-value of the gene enrichment was corrected by Benjamini–Hochberg FDR (false discovery rate).

## Supplementary information

**Additional file 1: Table S1.** Sample information for each horse (86 individuals) used in this study. **Table S2.** Number of SNPs for each breed. **Table S3.** Positively selected genes (top %1) identified by Fst method. **Table S4.** Overrepresented GO categories among genes showing high Fst values in DBP. **Table S5.** Positively selected genes identified by top 1% highest log2 (θπ·DBP/θπ·THB). **Table S6.** Gene functional enrichment categories showing high log2(θπ·DBP/θπ·THB) values in DBP. **Table S7.** Positively selected genes identified (top%1) by XP-CLR method. **Table S8.** Gene functional enrichment categories showing high XP-CLR values.

**Additional file 2: Figure S1**. Horse breeds used in this study. The figure was designed by the first author. **Figure S2**. Genome sequence depth for each horse in this study. **Figure S3.** Cross validation error (CV) plot from ADMIXTURE. **Figure S4**. Genomic landscape of positive selection signatures using the *F*_ST_ (A) and XP-CLR (B) values, between DBPs and all other horses.

## Data Availability

The whole genome sequencing data for all 17 individuals generated in this study have been deposited at NCBI SRA Database with accession code: PRJNA641243 or accessible through https://www.ncbi.nlm.nih.gov/bioproject/PRJNA641243. The experiment numbers are SRX8600443-SRX8600459. Additionally, other 69 horse genome samples that we downloaded from NCBI SRA Database (https://www.ncbi.nlm.nih.gov) are available under Bioproject numbers PRJNA430351, PRJNA277030, PRJNA400560, PRJEB14779, PRJNA168142, PRJNA184688, PRJNA288817, PRJNA291776, PRJNA306677 and PRJEB10854. Also, the NCBI accession numbers used in this study are found in Additional file 1: Table S1.

## Abbreviations

AMH: American miniature horse
BAM: Binary Alignment MAP
BWA: Burrows-Wheeler Aligner
XP-CLR: Cross population composite likelihood ratio
DBP: Debao pony
*F*_ST_: Fixation index
HNV: Hanoverian
HLS: Holsteiner
LD: Linkage disequilibrium
GATK: Genome Analysis Toolkit
GO: Gene ontology
GWAS: Genome-wide association studies
θπ: Nucleotide diversity
MNG: Mongolian horse
PCA: Principle component analysis
PSGs: positively selected genes
ROH: Runs of homozygosity
SNP: Single-nucleotide polymorphism
STB: Standardbred
THB: Thoroughbred
WGS: Whole genome sequencing
YKT: Yakutian

## References

[1] Darwin C (1868). The variation of animals and plants under domestication.

[2] Wang MS, Huo YX, Li Y, Otecko NO, Su LY, Xu HB, Wu SF (2016). Comparative population genomics reveals genetic basis underlying body size of domestic chickens. J Mol Cell Biol.

[3] Andersson L (2016). Domestic animals as models for biomedical research. Ups J Med Sci.

[4] Petersen JL, Mickelson JR, Cothran EG, Andersson LS, Axelsson J, Bailey E (2013). Genetic diversity in the modern horse illustrated from genome-wide SNP data. PLoS One.

[5] Brooks SA, Makvandi-Nejad S, Chu E, Allen JJ, Streeter C, Gu E, McCleery B, Murphy BA, Bellone R, Sutter NB (2010). Morphological variation in the horse: defining complex traits of body size and shape. Anim Genet.

[6] Makvandi-Nejad S, Hoffman GE, Allen JJ, Chu E, Gu E, Chandler AM, Loredo AI, Bellone RR, Mezey JG, Brooks SA, Sutter NB (2012). Four loci explain 83% of size variation in the horse. PLoS One.

[7] Mattern J (2010). Horses on the farm. Rourke Pub Group.

[8] Jiang Q, Wei Y, Huang Y, Jiang H, Guo Y, Lan G, Liao J (2011). The complete mitochondrial genome and phylogenetic analysis of the Debao pony (Equus caballus). Mol Biol Rep.

[9] Kader A, Li Y, Dong K, Irwan DM (2016). Population variation reveals independent selection toward small body size in Chinese Debao pony. Genome Biol Evol.

[10] Metzger J, Schrimpf R, Philipp U, Distl U (2013). Expression levels of LCORL are associated with body size in horses. PLoS One.

[11] Al-Abri MA, Posbergh C, Palermo K, Sutter NB, Eberth J, Hoffman GE, Brooks SA (2018). Genome-wide scans reveal a quantitative trait locus for withers height in horses near the ANKRD1 gene. J Equine Vet Sci.

[12] Dreger DL, Rimbault M, Davis BW, Bhatnagar A, Parker HG, Ostrander EA (2016). Whole-genome sequence, SNP chips and pedigree structure: building demographic profiles in domestic dog breeds to optimize genetic-trait mapping. Dis Model Mech.

[13] Weir BS, Cockerham CC (1984). Estimating F-statistics for the analysis of population structure. Evolution..

[14] Desai J, Shannon ME, Johnson MD, Ruff DW (2006). Nell1-deficient mice have reduced expression of extracellular matrix proteins causing cranial and vertebral defects. Hum Mol Gen.

[15] Shen J, James AW, Chung J, Lee K, Zhang JB, Ho S, Lee KS, Kim TM, Niimi T, Kuroda S, Ting K, Soo C (2012). NELL-1 promotes cell adhesion and differentiation via Integrinβ1. J Cell Biochem.

[16] Dateki S, Watanabe S, Kinoshita F, Yoshiura KI, Moriuchi H (2017). Identification of 11p14.1-p15.3 deletion probably associated with short stature, relative macrocephaly, and delayed closure of the fontanelles. Am J Med Genet A.

[17] Yamaguchi TP, Harpal K, Henkemeyer M, Rossant J (1994). FGFR-1 is required for embryonic growth and mesodermal patterning during mouse gastrulation. Genes Dev.

[18] Burke D, Wilkes D, Blundell TL, Malcolm S (1998). Fibroblast growth factor receptors: lessons from the genes. Trends Biochem Sci.

[19] Wang Q, Green RP, Zhao G, Ornitz DM (2001). Differential regulation of endochondral bone growth and joint development by FGFR1 and FGFR3 tyrosine kinase domains. Development..

[20] Su N, Jin M, Chen L (2014). Role of FGF/FGFR signaling in skeletal development and homeostasis: learning from mouse models. Bone Res.

[21] An B, Xia J, Chang T, Wang X, Xu L, Zhang L, Gao X, Chen Y, Li J, Gao H (2019). Genome-wide association study reveals candidate genes associated with body measurement traits in Chinese Wagyu beef cattle. Anim Genet.

[22] Bhatia M, Bonnet D, Wu D, Murdoch B, Wrana J, Gallacher L, Dick JE (1999). Bone morphogenetic proteins regulate the developmental program of human hematopoietic stem cells. J Exp Med.

[23] Seib FP, Lanfer B, Bornhauser B, Werner C (2010). Biological activity of extracellular matrix-associated BMP-2. J Tissue Eng Regen Med.

[24] Kugimiya F, Kawaguchi H, Kamekura S, Chikuda H, Ohba S, Yano F, Ogata N, Katagiri T, Harada Y, Azuma Y, Nakamura K, Chung UI (2005). Involvement of endogenous bone morphogenetic protein (BMP) 2 and BMP6 in bone formation. J Biol Chem.

[25] Tan TY, Gonzaga-Jauregui C, Bhoj EJ (2017). Monoallelic BMP2 variants predicted to result in Haploinsufficiency cause craniofacial, skeletal, and cardiac features overlapping those of 20p12 deletions. Am J Hum Genet.

[26] Lee KY, Singh MK, Ussar S (2015). Tbx15 controls skeletal muscle fibre-type determination and muscle metabolism. Nat Commun.

[27] Zhang L, Ma X, Xuan J, Wang H, Yuan Z, Wu M, Liu R, Zhu C, Wei C, Zhao F, Du L (2016). Identification of MEF2B and TRHDE gene polymorphisms related to growth traits in a new Ujumqin sheep population. PLoS One.

[28] Yang X-Q, Guo L-J, Zhai C-Y, Yu H, Liu H, Liu D (2009). Expression, characterization, and variation of the porcine calpain 7 gene. Acta Agric Scand Sect A Anim Sci.

[29] Monda KL, Chen GK, Taylor KC (2013). A meta-analysis identifies new loci associated with body mass index in individuals of African ancestry. Nat Genet.

[30] Shin S, Heo J, Yeo J, Lee C, Chung E (2012). Genetic association of phosphodiesterase 1B (PDE1B) with carcass traits in Korean cattle. Mol Biol Rep.

[31] Zhang X, Chu Q, Guo G (2017). Genome-wide association studies identified multiple genetic loci for body size at four growth stages in Chinese Holstein cattle. PLoS One.

[32] Tanaka KI, Xue Y, Nguyen-Yamamoto L, Morris JA, Kanazawa I, Sugimoto T, Wing SS, Richards JB, Goltzman D (2018). FAM210A is a novel determinant of bone and muscle structure and strength. Proc Natl Acad Sci U S A.

[33] Tang Z, Liang R, Zhao S, Wang R, Huang R, Li K (2014). CNN3 is regulated by microRNA-1 during muscle development in pigs. Int J Biol Sci.

[34] Wang J, Li ZJ, Lan XY, Hua LS, Huai YT, Huang YZ, Ma L, Zhao M, Jing YJ, Chen H, Wang JQ (2010). Two novel SNPs in the coding region of the bovine PRDM16 gene and its associations with growth traits. Mol Biol Rep.

[35] Wood TL, Streck RD, Pintar JE (1992). Expression of the IGFBP-2 gene in post-implantation rat embryos. Development.

[36] Koltes JE, Mishra BP (2009). A nonsense mutation in cGMP-dependent type II protein kinase (PRKG2) causes dwarfism in American Angus cattle. Proc Natl Acad Sci U S A.

[37] Gudbjartsson DF, Walters GB, Thorleifsson G (2008). Many sequence variants affecting diversity of adult human height. Nat Genet.

[38] Yang M, Fu J, Lan X, Sun Y, Lei C, Zhang C, Chen H (2013). Effect of genetic variations within the SH2B2 gene on the growth of Chinese cattle. Gene..

[39] Deng MT, Zhu F, Yang YZ, Yang FX, Hao JP, Chen SR, Hou ZC (2019). Genome-wide association study reveals novel loci associated with body size and carcass yields in Pekin ducks. BMC Genomics.

[40] Yasukochi Y, Sakuma J, Takeuchi I, Kato K, Oguri M, Fujimaki T, Horibe H, Yamada Y (2018). Identification of three genetic variants as novel susceptibility loci for body mass index in a Japanese population. Physiol Genomics.

[41] Nolte W, Thaller G, Kuehn C (2019). Selection signatures in four German warmblood horse breeds: tracing breeding history in the modern sport horse. PLoS One.

[42] Moon S, Lee JW, Shin D, Shin KY, Kim J, Choi IY (2015). A genome-wide scan for selective sweeps in racing horses. Asian Austral J Anim.

[43] Metzger J, Karwath M, Tonda R (2015). Runs of homozygosity reveal signatures of positive selection for reproduction traits in breed and non-breed horses. BMC Genomics.

[44] Gori F, Thomas T, Hicok KC, Spelsberg TC, Riggs BL (1999). Differentiation of human marrow stromal precursor cells: bone morphogenetic protein-2 increases OSF2/CBFA1, enhances osteoblast commitment, and inhibits late adipocyte maturation. J Bone Miner Res.

[45] Kuroda S, Tanizawa K (1999). Involvement of epidermal growth factor-like domain of NELL proteins in the novel protein–protein interaction with protein kinase C. Biochem Biophys Res Commun.

[46] Qi H, Kim JK, Ha P, Chen X, Chen E, Chen Y (2019). Inactivation of Nell-1 in chondrocytes significantly impedes appendicular Skeletogenesis. J Bone Miner Res.

[47] James AW, Chiang M, Asatrian G (2016). Vertebral implantation of NELL-1 enhances bone formation in an osteoporotic sheep model. Tissue Eng Part A.

[48] Lango Allen H, Estrada K, Lettre G, Berndt SI, Weedon MN (2010). Hundreds of variants clustered in genomic loci and biological pathways affect human height. Nature..

[49] Sutter NB, Bustamante CD, Chase K, Gray MM, Zhao K, Zhu L, Padhukasahasram B (2007). A single IGF1 allele is a major determinant of small size in dogs. Science..

[50] Pryce JE, Hayes BJ, Bolormaa S, Goddard ME (2011). Polymorphic regions affecting human height also control stature in cattle. Genetics..

[51] Signer-Hasler H, Flury C, Haase B, Burger D, Simianer H, Leeb T, Rieder S (2012). A genome-wide association study reveals Loci influencing height and other conformation traits in horses. PLoS One.

[52] Tetens J, Widmann P, Kühn C, Thaller G (2013). A genome-wide association study indicates LCORL/NCAPG as a candidate locus for withers height in German Warmblood horses. Anim Genet.

[53] Staiger EA, Al Abri MA, Pflug KM, Kalla SE, Ainsworth DM, Miller D, Raudsepp T, Sutter NB, Brooks SA (2016). Skeletal variation in Tennessee walking horses maps to the LCORL/NCAPG gene region. Physiol Genomics.

[54] Sevane N, Dunner S, Boado A, Cañon J (2016). Polymorphisms in ten candidate genes are associated with conformational and locomotive traits in Spanish purebred horses. J Appl Genetics.

[55] Junior AB, Quirino CR, Vega WHO, Rua MAS, David CMG, Jardim JG (2018). Polymorphisms in the LASP1 gene allow selection for smaller stature in ponies. Livest Sci.

[56] Ma Y, Ding X, Qanbari S, Weigend S, Zhang Q, Simianer H (2015). Properties of different selection signature statistics and a new strategy for combining them. Heredity..

[57] Li H, Durbin R (2009). Fast and accurate short read alignment with burrows-wheeler transform. Bioinformatics..

[58] Li H, Handsaker B, Wysoker A, Fennell T, Ruan J, Homer N, Marth G, Abecasis G, Durbin R (2009). 1000 genome project data processing subgroup. The sequence alignment/map format and SAMtools. Bioinformatics..

[59] McKenna A, Hanna M, Banks E, Sivachenko A, Cibulskis K, Kernytsky A, Garimella K, Altshuler D, Gabriel S (2010). The genome analysis toolkit: a MapReduce framework for analyzing next-generation DNA sequencing data. Genome Res.

[60] Yang J, Lee SH, Goddard ME, Visscher PM (2011). GCTA: a tool for genome-wide complex trait analysis. Am J Hum Genet.

[61] Purcell S, Neale B, Todd-Brown K, Thomas L, Ferreira MA, Bender D, Maller J, Sklar P, de Bakker PI, Daly MJ, Sham PC (2007). PLINK: a tool set for whole-genome association and population-based linkage analyses. Am J Hum Genet.

[62] Alexander DH, Novembre J, Lange K (2009). Fast model-based estimation of ancestry in unrelated individuals. Genome Res.

[63] Lawson DJ, Hellenthal G, Myers S, Falush D (2012). Inference of population structure using dense haplotype data. PLoS Genet.

[64] Chen H, Patterson N, Reich D (2010). Population differentiation as a test for selective sweeps. Genome Res.

[65] Reimand J, Arak T, Vilo J (2011). G: profiler--a web server for functional interpretation of gene lists (2011 update). Nucleic Acids Res.

