# Supplementary material for: Comparative population genomic analysis uncovers novel genomic footprints and genes associated with small body size in Chinese pony
Source: BMC Genomics. 2020 Jul 20;21:496. doi: 10.1186/s12864-020-06887-2 (PMC7370493; doi:10.1186/s12864-020-06887-2)

**Fig. S1**: Horse breeds used in this study. The figure was designed by the first author.

**
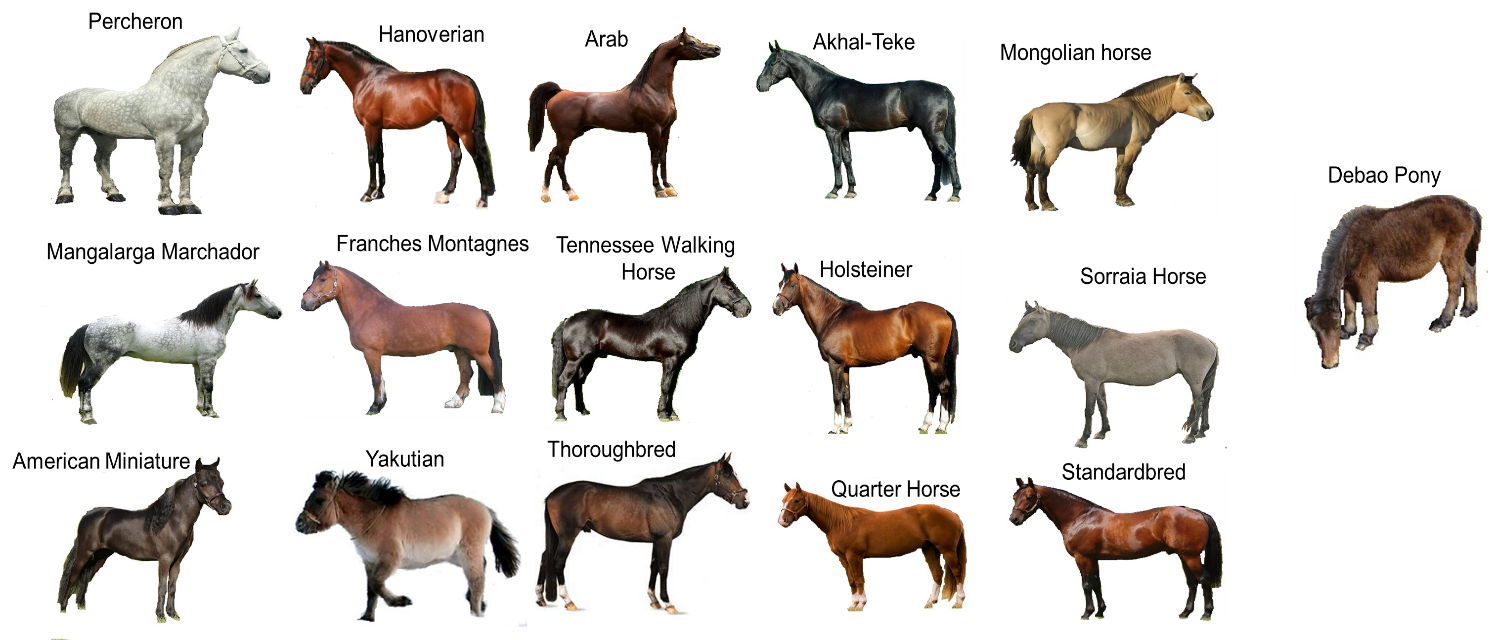
**

**Fig. S2**: Genome sequence depth for each horse in this study


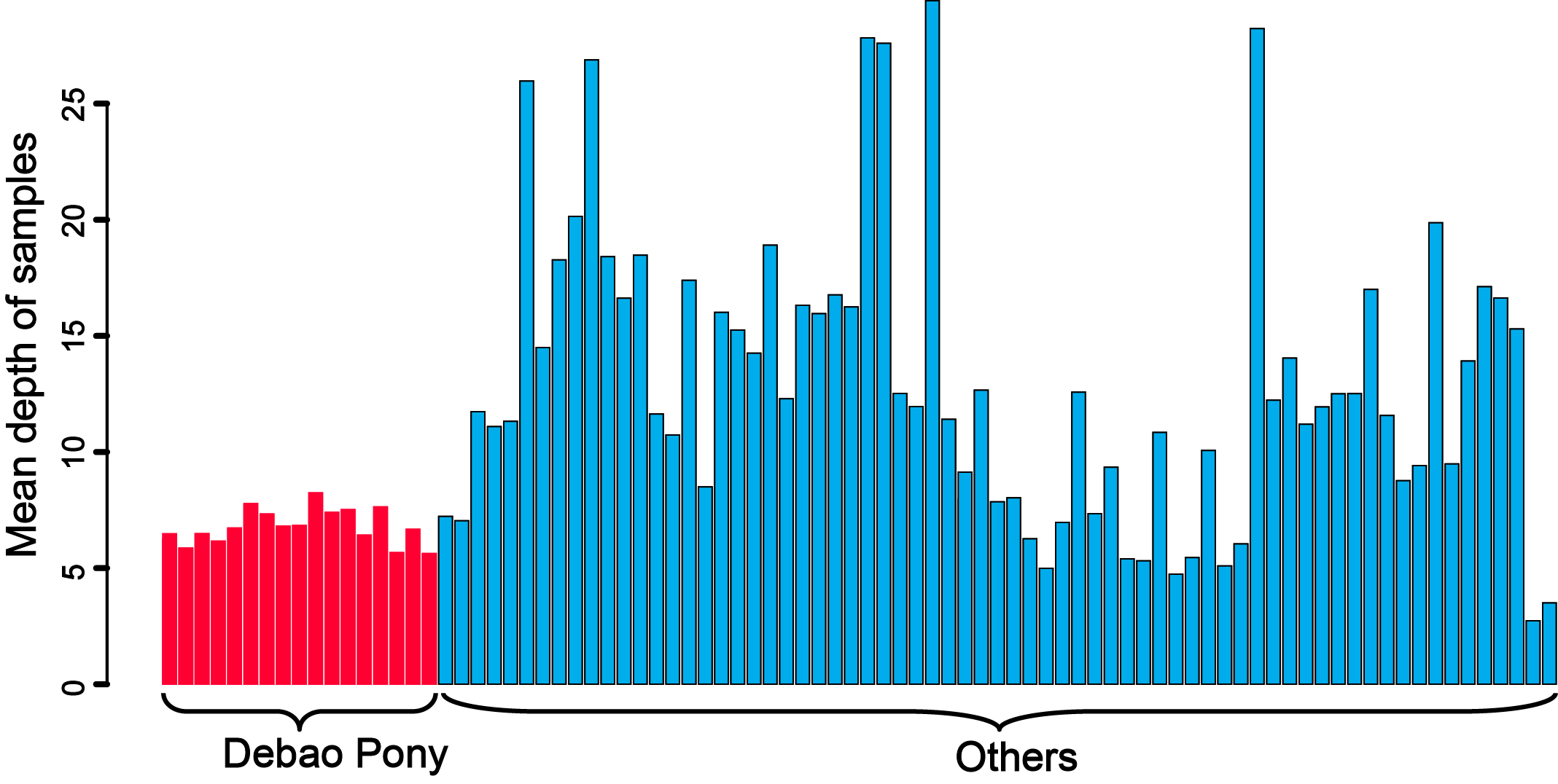


**Fig. S3:** Cross validation error (CV) plot from ADMIXTURE.


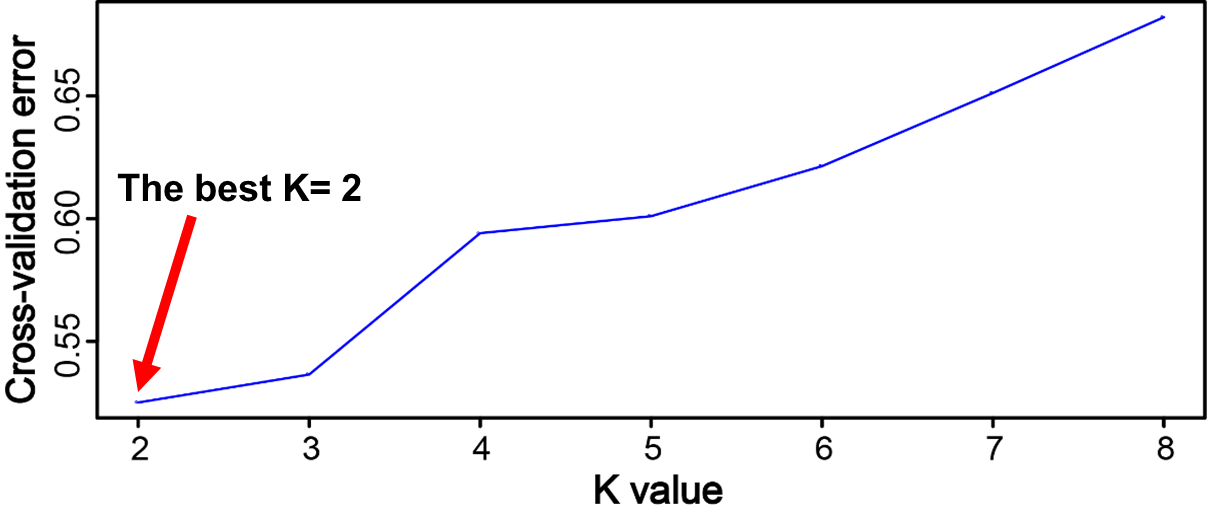


**Fig. S4**: Genomic landscape of positive selection signatures using the *F*_ST_ (A) and XP-CLR (B) values, between DBPs and all other horses.


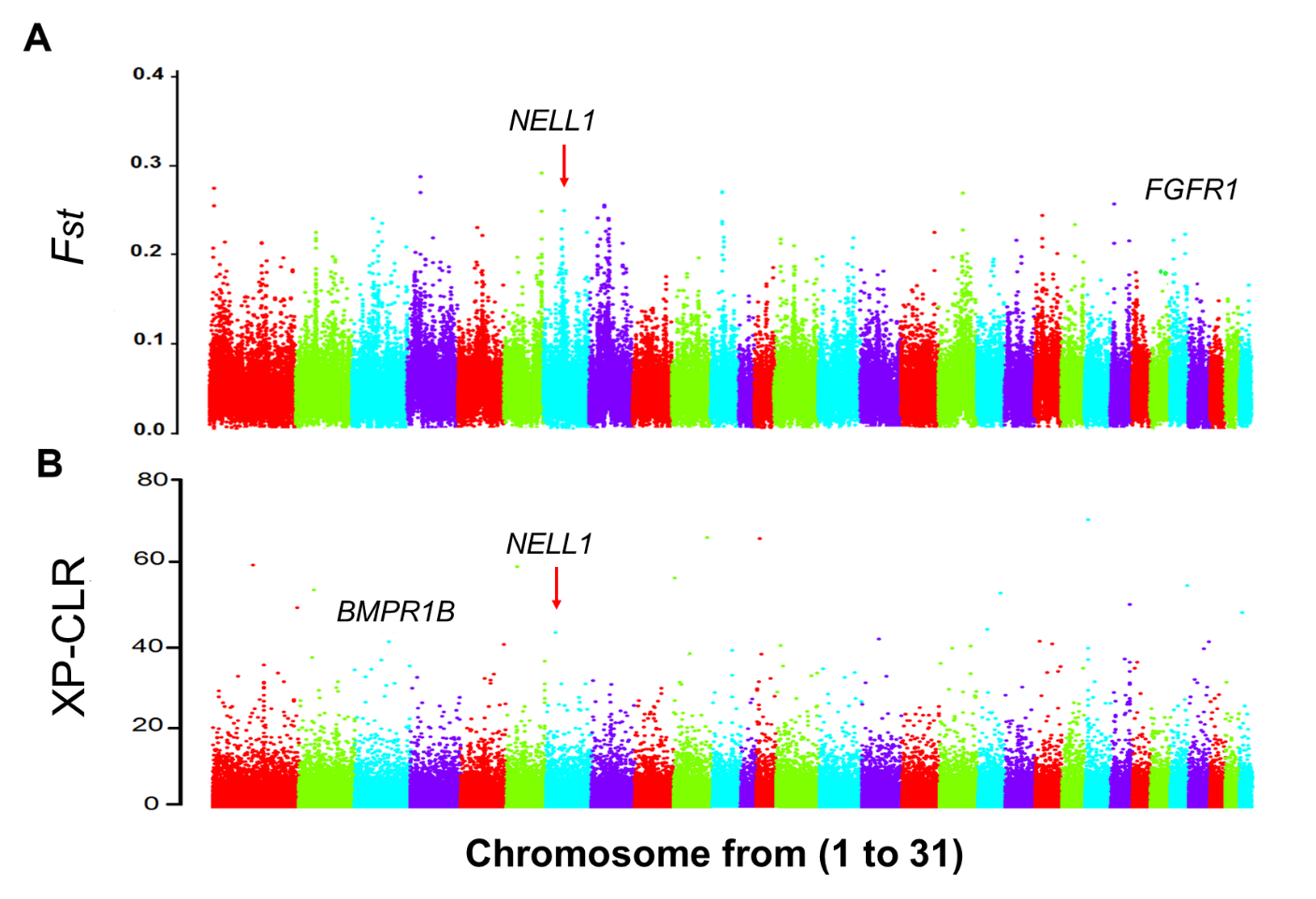

Supplement: Supplementary file 2 — Additional file 2: Figure S1. Horse breeds used in this study. The figure was designed by the first author. Figure S2. Genome sequence depth for each horse in this study. Figure S3. Cross validation error (CV) plot from ADMIXTURE. Figure S4. Genomic landscape of positive selection signatures using the FST (A) and XP-CLR (B) values, between DBPs and all other horses. [file 12864_2020_6887_MOESM2_ESM.docx]
